# Behavioral Markers in Older Adults During COVID-19 Confinement: Secondary Analysis of In-Home Sensor Data

**DOI:** 10.2196/56678

**Published:** 2025-06-02

**Authors:** Knoo Lee, Noah Marchal, Erin L Robinson, Kimberly R Powell

**Affiliations:** 1 Sinclair School of Nursing, University of Missouri, 915 Hitt St, S413, Columbia, MO, 65212, United States, 1 573-884-0421; 2 Institute for Data Science and Informatics, University of Missouri, Columbia, MO, United States; 3 College of Health Professions, University of Missouri, Columbia, MO, United States

**Keywords:** older adults, aging-in-place, community dwelling, sensors, healthy lifestyle

## Abstract

**Background:**

Older adults were disproportionately affected by the COVID-19 pandemic, with a high number of deaths occurring in this age group. The impact of social isolation and home confinement continues to impact the mental and emotional health of older adults, despite the end of the COVID-19 pandemic. Unhealthy lifestyle behaviors, including physical and social inactivity, and poor sleep quality, have been reported. Recommendations for healthy lifestyle changes have primarily targeted the general population, highlighting the need for personalized recommendations for vulnerable older adults. Remote sensing technologies may offer an opportunity to understand behavior changes among older adults and provide personalized recommendations.

**Objective:**

This study aims to describe the effects of home confinement and social isolation on community-dwelling older adults during the COVID-19 outbreak and investigate how integrated computing technologies, such as remote sensors installed in homes, can help inform recommendations for safe and healthy lifestyles.

**Methods:**

As part of a larger study and ongoing research with community-dwelling older adults, remote sensors including bed transducers, 3D depth cameras, and passive infrared (PIR) motion sensors were installed in the homes of the study sample. We compared features derived from sensors for approximately one month before the COVID-19 outbreak (January 14, 2020-February 13, 2020) and one month after the onset of the pandemic (March 14, 2020-April 13, 2020). We used descriptive statistics and paired-sample *t* tests to compare the 2 time periods, pre-COVID-19 and early-COVID-19.

**Results:**

Sensor data from 64 older adults were analyzed, the majority identifying as female (n=51, 80%), aged >76 years (n=58, 92%), and living alone (n=50, 78%). Results from paired-sample *t* tests demonstrated significant differences in sensor features between the pre–COVID-19 and early–COVID-19 time periods. We found statistically significant differences in bed restlessness (pre-COVID: mean 14.98, SD 5.10; early-COVID: mean 15.56, SD 5.25; *t*_554_=−4.10; *P*<.001), time spent in bed (pre-COVID: mean 32,547.41, SD 9269.96; early-COVID: mean 33,494.73, SD 10,887.33; *t*_554_=−2.81; *P*=.005), pulse (pre-COVID: mean 68.45, SD 3.30; early-COVID: mean 68.10, SD 3.36; *t*_554_=3.66; *P*<.001), respiration (pre-COVID: mean 14.54, SD 1.32; early-COVID: mean 14.41, SD 1.31; *t*_553_=3.72; *P*<.001), and stride length (pre-COVID: mean 29.10, SD 4.813; early-COVID: mean 28.76, SD 5.016; *t*_595_=2.17; *P*=.03). Among the study sample, bed restlessness and time spent in bed increased between the 2 time periods, while pulse, respiration, and stride length decreased.

**Conclusions:**

This study highlights that home confinement during the pandemic significantly impacted the behavior and health of older adults, leading to more sedentary lifestyles and poorer sleep quality. These changes may contribute to a decline in physical and mental health, increasing the risk of depression, lack of social contact, and diminished functional capacity. The findings underscore the need for older adults in future infectious disease outbreaks and suggest in-home sensor technology as a potential tool for monitoring their health and guiding decisions during periods of confinement.

## Introduction

Nearly 75% of COVID-19–linked deaths (more than 630,000) between 2020 and 2022 occurred among people of 65 years and older [1]. This suggests that COVID-19 has taken the lives of nearly 1 in 100 US older adults in just over 2 years [2]. Although case demographics of COVID-19 in the United States fluctuated over time and between the US states since the beginning of the pandemic, older adults were affected disproportionately in terms of hospitalizations and deaths [3]. To limit the spread of COVID-19, lockdowns with home confinement and social distancing measures were implemented by public health authorities around the world. Despite being recognized as effective measures to curb the spread of the COVID-19 outbreak, social distancing and home confinement caused profound disruption of normal lifestyles, exacerbating mental health concerns, and social isolation among older adults.

The home confinement and social distancing measures implemented to reduce the spread of COVID-19 during the COVID-19 outbreak resulted in significant negative effects on mental health. Studies have reported worsening mental health symptoms, including stress, anxiety, depression, insomnia, and intrusive thinking [4,5] among older adults as a result of pandemic-induced social isolation. In addition, these psychosocial symptoms have been found to be significantly associated with unhealthy lifestyle behaviors. Ammar et al [6], found that the COVID-19 home confinement resulted in increased physical and social inactivity, poor sleep quality, and unhealthy diet. Community-dwelling older adults who lived alone reported struggles with eating properly during the early stages of the pandemic [7]. It is also recognized that lifestyle behaviors, including physical inactivity, unhealthy diets, and poor sleep quality, significantly affect the severity of COVID-19 and can extend recovery time [8]. This illustrates the cyclical nature of home confinement, where measures taken to minimize COVID-19 infection risk inadvertently contribute to factors such as reduced physical activity, which in turn can lead to negative mental health outcomes and worsen overall COVID-19 impacts. These negative impacts during the COVID-19 lockdown were observed not only among older adults but also among elite athletes, underscoring the widespread adverse effects of home confinement across different populations [9]. Results from a worldwide cohort study revealed that older adults were more likely to experience weight gain, physical inactivity, and perceived frailty during the COVID-19 lockdown [10].

Alleviating the risk factors associated with negative mental health symptoms experienced by older adults is a high priority. However, available recommendations from organizations including the World Health Organization (WHO), American Heart Association (AHA), and American College of Sports Medicine (ACSM), mainly target the general population [11-13]. To move forward, it is imperative to consider the holistic behavioral patterns among older adults. This understanding forms the foundational groundwork for crafting personalized recommendations, which hinge on precise user profiling and the subsequent dissemination of these recommendations to the individuals themselves.

This study is uniquely positioned to examine the physiological and behavioral patterns of community-dwelling older adults during the onset of the COVID-19 pandemic. At this time, participants were already recruited into a larger, ongoing, observational study that installed embedded sensing technologies in their homes. The goal of that project was to develop a tailored interface for older adults and their family caregivers to monitor the older adults’ health-generated sensor data. Since the sensor technologies were already in place before the onset of the COVID-19 pandemic, they offer us a real-time glimpse of community-dwelling older adults’ patterns during the initial onset of a global pandemic. The purpose of this study was to describe the effects of home confinement and social isolation on community-dwelling older adults during the COVID-19 outbreak. By analyzing data derived from in-home sensor systems of older adults, we intend to inform recommendations for safe and healthy lifestyles during future infectious disease outbreaks.

## Methods

### Recruitment

Residents (n=143) were recruited from Americare Senior Living facilities located in mid-Missouri between June 2017 to July 2022. For this analysis, we selected residents (n=64) who had at least one of 3 types of sensor data (bed, gait, and motion) for the duration of the study time period (January 14, 2020-April 13, 2020).

### Procedures

A suite of in-home monitoring sensors, developed at the Center to Stream Healthcare In-Place, and commercially licensed by Foresite Healthcare, LLC [14,15], were installed within the study participants’ homes. Data were collected from 3 types of in-home sensors: a bed mat containing 4 hydraulic transducers that captured ballistocardiogram signals, a thermal depth sensor for capturing gait and detecting falls, and passive infrared (PIR) motion sensors [16-19]. The hydraulic bed mat was installed directly beneath the side of the bed that a participant sleeps on, minimizing the amount of error introduced by companions or pets. Errors in gait measurements are reduced by using a probability density function model to identify participant walks separate from those of visitors or caregivers. However, it is understood that the motion sensors capture resident-level activity throughout the home. The raw data were first preprocessed algorithmically to derive the individual health parameters using existing methods [17,20-24], with the exception of percent restless and walk density, which were calculated respectively as the percent of time in bed spent restless and the number of walks recorded per day. The raw ballistocardiogram waveforms were sampled and collected at 100 hertz (Hz), from which the pulse rate, respiration, and restlessness parameters were extracted using signal processing algorithms [17]. The resolution of the depth sensor allows for collecting gait parameters during purposeful walks at 15 frames per second [22], as well as detecting falls. The motion sensor firings were collected in 7-second intervals over the duration from the onset of detected motion [25,26]. To account for the differing sampling rates, we aggregated the derived health parameters as daily averages before analysis. Due to the heterogeneity of participant home environments not all possible sensor variables were collected from participants. Bed mats were not installed for participants who sleep in hospital beds with adjustable height or in a recliner chair. Gait parameters were not collected from the thermal depth sensor for participants who are wheelchair-bound. In addition, Zigbee motion sensors required frequent battery changes every 6‐8 weeks, which reduced the availability for the period during which facility visitation was limited after the onset of the pandemic. In order to compare the periods before and after the shelter-in-place order took effect, we limited our participant cohort to only those with data both before and immediately after. All 64 participants initially recruited were included in at least one of the 3 sensor analyses (bed, gait, or motion). However, not all participants had complete data for every sensor type. In specific, 43 participants had complete bed sensor data, 49 participants had complete gait sensor data, and 43 participants had complete motion sensor data. Participants were categorized based on the availability of usable data for each sensor type. Some participants contributed to more than one category, while others contributed to only one. For example, a participant with complete gait sensor data but no bed or motion sensor data was included in the gait analysis but not in the others. Importantly, no participants were excluded entirely, as all 64 provided usable data for at least one sensor type. This aligns with the inclusion criterion of having at least one form of sensor data during the study period. There were 14 individuals with roommates and 50 without roommates. Gait observations were filtered using Gaussian distributions of stride time, stride length, and walking speed, relative to participant height, to form clusters. These clusters were validated using a probability density function, effectively removing outliers caused by visitors’ gait metrics [27].

Motion sensors capture room-level activity within the residence, including movement from visitors, family members, and facility staff. The data is calculated as the number of 7-second intervals during which motion is detected. Since our focus is on the overall patterns between the periods immediately before and after the onset of the pandemic, we consider these motion variables as indicators of isolation within the residence rather than as measurements of individual behavior.

### Statistical Analysis

We compared 17 health parameters (Table 1) derived from sensors installed in apartments of older adults who resided in an independent living community pre- and early-COVID-19 outbreak. Descriptive statistics and paired-sample *t* tests for each health parameter were calculated between the one-month pre–COVID-19 and post–COVID-19 periods. The pre–COVID-19 period represented approximately one month before the Centers for Medicare and Medicaid Services (CMS) issuance of a mandatory stay at home order (January 14, 2020-February 13, 2020) and the early–COVID-19 period represented approximately one month after the stay-at-home order (March 14, 2020-April 13, 2020) [28]. The *t* tests were conducted in Python using Scipy.stats package’s ttest_rel function with null observations omitted. We used group-level data for *t* tests. There was not a treatment, outcome effect measured, or comparison against control group involved in the analysis that would lead to confound or performance bias. Gait data is filtered to the resident with a probability density model to prevent noise introduced by walks captured by apartment visitors. We used only the available data without applying any imputation techniques for missing entries. Participants with complete data for both the pre- and post-COVID lockdown periods were included. If postlockdown data were missing, the participant was excluded from the *t* test analysis to maintain methodological consistency.

**Table 1.** Variables included in the analysis.

Variable	Description	Unit
Average pulse rate	Average pulse rate computed with energy algorithm	Per minute
Minimum pulse rate	Minimum pulse rate computed with energy algorithm	Per minute
Maximum pulse rate	Maximum pulse rate computed with energy algorithm	Per minute
Average respiration rate	Average respiration rate	Per minute
Maximum respiration rate	Maximum respiration rate	Per minute
Restlessness	Number of seconds of restlessness in bed	Count
Time in bed	Number of seconds in bed	Count
Percentage of restlessness	Ratio of seconds_restless to seconds_in_bed * 100	Percent
Height	Walking height	Inches
Walking speed	Walking speed	Centimeters per second
Stride time	Time taken for stride	Seconds
Stride length	Length of stride	Centimeters
Walk density	Count of walks	Count
Bathroom motion	Motion hits bathroom	Count
Bedroom motion	Motion hits bedroom	Count
Front door motion	Motion hits front door	Count
Living room motion	Motion hits living room	Count

### Ethical Considerations

Participants consented through the study protocol approved by the University of Missouri Institutional Review Board for a National Library of Medicine award (R01LM012221) entitled “Linguistic Summarization of Sensor Data for Early Illness Recognition in Eldercare.” The study was approved by the institutional review board of an R1 university. Written informed consent was obtained from all participants, and the original consent included provisions for the future use of anonymized data in secondary analyses. All data were deidentified before analysis, and strict measures were implemented to protect participant information, including secure storage and encryption. Data transmission adhered to Health Insurance Portability and Accountability Act requirements. Participants were not compensated for their involvement in this study.

## Results

### Participant Characteristics

There were 64 participants (See Table 2 for baseline characteristics), of whom 51 (80%) were female and 13 (20%) were male. Participants were grouped by age as follows: 1 (2%) was under 65 years old, 5 (8%) were between 66 and 75, 24 (38%) were between 76 and 85, 26 (41%) were between 86 and 95, and 8 (13%) were older than 95 years. A total of 14 participants (22%) reported using a wheelchair, while 55 (86%) reported using a walker. A total of 14 (22%) participants reported having a roommate, while 50 (78%) did not.

**Table 2.** Participant characteristics (N=64).

Baseline characteristics	Value, n (%)	
Sex		
Female	51 (80)	
Male	13 (20)	
Age (years)		
<65	1 (2)	
66‐75	5 (8)	
76‐85	24 (38)	
86‐95	26 (41)	
>95	8 (13)	
Mobility aids		
Wheelchair		
Yes	14 (22)	
No	50 (78)	
Walker		
Yes	55 (86)	
No	9 (14)	
Has roommate		
Yes	14 (22)	
No	50 (78)	

### Statistical Outcomes

Group-level paired *t* test revealed statistically significant differences in pulse, respiration, restlessness, stride, walk speed, and times spent in bed between pre– and early–COVID-19 time periods (See Table 3).

**Table 3.** Paired *t* test results.

Variables	Pre-COVID-19, mean (SD)		Early-COVID-19, mean (SD)		*t* test (*df*)		*P* value	Cohen *d*
Bathroom	351.42 (305.82)		309.45 (275.82)		5.19 (1314)		<.001	0.144
Bedroom	499.57 (454.22)		497.95 (394.52)		0.17 (1314)		.87	0.004
Front door	121.34 (158.36)		134.07 (159.46)		−2.91 (1314)		.004	−0.08
Living room	431.11 (439.04)		371.4 (393.48)		5.14 (1314)		<.001	0.143
Pulse (mean)	68.45 (3.3)		68.1 (3.36)		3.66 (554)		<.001	0.107
Pulse (max)	88.61 (5.99)		88.29 (5.71)		1.28 (554)		.20	0.055
Pulse (min)	51.43 (3.51)		51.4 (3.72)		0.29 (554)		.78	0.01
Respiration(mean)	14.54 (1.32)		14.41 (1.31)		3.72 (553)		<.001	0.099
Respiration (max)	18.98 (0.75)		18.95 (0.81)		0.64 (554)		.52	0.031
In bed (s)	32,547.41 (9269.96)		33,494.73 (10,887.33)		−2.81 (554)		.005	−0.094
Restless (%)	14.98 (5.1)		15.56 (5.25)		−4.1 (554)		<.001	−0.111
Restless (s)	4790.82 (1876.29)		5223.73 (2376.45)		−6.15 (554)		<.001	−0.204
Height	63.75 (3.75)		63.37 (4.17)		4.38 (611)		<.001	0.095
Stride length	29.1 (4.81)		28.76 (5.02)		2.17 (595)		.03	0.071
Stride time	1.45 (0.25)		1.51 (0.3)		−4.94 (595)		<.001	−0.193
Walk speed	21.81 (5.27)		21.33 (5.79)		2.79 (611)		.006	0.086
Walk density	103.16 (87.75)		107.91 (111.56)		−1.24 (611)		.22	−0.048

Regarding monthly activity by room locations, there was a significant decrease in levels of detected motions in the bathroom and living room during early–COVID-19 (bathroom: mean 309.45, SD 275.82; *t*_1314_=5.19; *P*<.001; living room: mean 371.40, SD 393.48; *t*_1314_=5.14; *P*<.001) compared with pre–COVID-19 (bathroom: mean 351.42, SD 305.82; living room: mean 431.11, SD 439.04). The movement at the front door, on the other hand, significantly increased during early–COVID-19 (mean 134.07, SD 159.46; *P*=.004) compared with the pre–COVID-19 time period (mean 121.34, SD 158.36).

Regarding changes in walk-related measurements, stride time increased (pre-COVID: mean 1.45, SD 0.25; early-COVID: mean 1.51, SD 0.30; *t*_595_=−4.94; *P*<.001), while stride length (pre-COVID: mean 29.104, SD 4.813; early-COVID: mean 28.756, SD 5.016; *t*_595_=2.167; *P*=.03), walk speed (pre-COVID: mean 21.81, SD 5.27; early-COVID: mean 21.33, SD 5.79; *t*_611_=2.79; *P*=.006), and height (pre-COVID: mean 63.75, SD 3.75; early-COVID: mean 63.37, *SD* 4.17, *t*_611_=4.38; *P*<.001) decreased.

Other measurements with significant differences include restlessness (increase; pre-COVID: mean 14.98, SD 5.10; early-COVID: mean 15.56, SD 5.25; *t*_554_=−4.10; *P*<.001), seconds in bed (increase; pre-COVID: mean 32,547.41, SD 9269.96; early-COVID: mean 33,494.73, SD 10,887.33, *t*_554_=−2.81; *P*=.005), pulse (decrease; pre-COVID: mean 68.45, SD 3.30; early-COVID: mean 68.10, SD 3.36; *t*_554_=3.66; *P*<.001), and respiratory rate (decrease; pre-COVID: mean 14.54, SD 1.32; early-COVID: mean 14.41, SD 1.31; *t*_553_=3.72; *P*<.001).

## Discussion

### Principal Findings

Our findings provide evidence on the impact of home confinement and social distancing measures on the behavior and health of aging in place older adults. When we compared pre–COVID-19 to the early–COVID-19 time periods, participants in our study showed a significant decrease in motion detected in the bathroom and living room, while motion at the front door increased. There was a significant decrease in stride, walk speed, and height; conversely, stride time increased. Sensors detected an increase in in-bed restlessness and a decrease in both average pulse and average respiratory rate. These findings suggest the decline in activity during home confinement could negatively impact the physical and mental health of older adults.

### Comparison With Previous Work

Regarding activities by location, the sedentary lifestyle that older adults exhibit in the living room may have long-term negative effects on their physical health. Previous research demonstrated a link between prolonged sitting and increased risk of obesity, cardiovascular disease, and type 2 diabetes [29-31]. These studies highlight the importance of promoting physical activity and reducing sedentary habits among older adults, further supporting the utility of remote sensors in discovering early signs of physical inactivity or sedentary lifestyles. Despite the lower priority assigned to the mentioned health risks (obesity, cardiovascular disease, type 2 diabetes) in comparison to safeguarding and quarantine due to the risks and severity of COVID-19 infection during the pandemic, it is essential to highlight that addressing the issue of physical inactivity becomes even more crucial in these challenging circumstances necessitating home confinement. The decline in bathroom usage may impact a number of health issues, including urinary tract infections, constipation, dehydration, and kidney stones [32-34]. In addition, this pattern of decreased bathroom use may be due to residents not performing hygiene tasks such as getting dressed, combing hair, etc. These problems could lead to more serious complications for older adults, who might have additional health issues or mobility issues [7]. Early detection of changes in bathroom use could alert caregivers before the occurrence of a significant health event. Finally, the increase in activity at the front door may indicate a rise in both residents and building service providers, such as building managers and caregivers, frequenting that area to help support residents meeting a variety of needs (eg, retrieve meals, send mail, wellness, and vital checks). This could be attributed to the surge in the use of food delivery services following the stay-at-home orders during the COVID-19 pandemic [35,36].

Regarding the gait parameters, our findings of increased height and stride time, and decreased walk speeds could suggest a decline in physical activity and mobility, which can have negative effects on overall health and well-being. According to a study [37] examining the impact of COVID-19 on older adults using the Kihon checklist regarding physical activity, a significant decline in total physical activity time for all frailty categories was reported. The decline in physical activity, which aligns with our study’s findings of decreased stride length and walk speeds, suggests that the confinement during the pandemic may have had a negative effect on the physical activity levels of older adults.

Our findings of increased restlessness and seconds spent in bed may be an indication of poor sleep quality, which can lead to negative mental health outcomes, including depression. As opportunities for socialization diminished, older adults spent more time in bed as they were adjusting to a more confined lifestyle. In a study [38], the researchers explored the relationship between social isolation, depression, and the well-being of older adults. Their findings revealed that older adults who experienced social isolation had a greater likelihood of developing depression and anxiety. In addition, A recent report indicates that physical exercise helped promote mental well-being while reducing the severity of acute respiratory infection symptoms and the number of symptom days (−2.24 d) during the follow-up period [39]. Findings from these studies further support that the social isolation resulting from the COVID-19 pandemic could be associated with symptoms of depression [35].

In examining the decline in average pulse and respiratory rates in our findings, research [40,41] shows that reduced physical activity, particularly in older adults with high-risk or cardiovascular disease, was a widespread consequence of lockdowns, leading to lower metabolic rates and decreased cardiovascular output, which could explain the drop in vitals such as heart and respiratory rates. However, the anticipated increase in stress-induced vitals, due to the pandemic’s mental health toll, is not consistently reflected in physiological markers. While anxiety and depression rates among older adults did increase during COVID-19, research shows the factors such as older adults’ emotional resilience and ability to manage stress may suggest that these psychological stressors do not consistently lead to elevated pulse or respiratory rates [42]. The physiological response to stress during quarantine may have been modulated by factors such as extended rest periods and reduced physical activity. Future research should investigate the complex interplay between stress, medication use, and physiological markers while also examining the long-term health implications of sedentary behavior in older adults. This insight is particularly relevant during prolonged periods of isolation or crisis, as it can inform policymakers in the development of remote health monitoring systems aimed at supporting the physical and mental well-being of elderly populations.

The variability of movement shown in the bedroom among participants, indicated by statistical insignificance in their movement patterns (*P*=.87), suggests that while home confinement significantly affects movement patterns mentioned above, individual responses can vary greatly. This variability highlights the need for personalized health care strategies, as home confinement impacts different people in different ways. For example, differences in movement patterns may be influenced by factors such as biological sex, age, or pre-existing health conditions. A recent study highlights how these demographics, such as biological sex and social determinants of health, can affect the psychological impact during home confinement [43]. Similarly, another systematic review on physical activities during home confinement points out the need for tailored recommendations for vulnerable populations, including older adults, further supporting the need for precision health care approaches [44].

Using in-home sensors for aging-in-place older adults can provide insights into their behavioral patterns. Moreover, a significant number of older adults prefer to remain in their home environment rather than move into institutional settings like nursing homes. The use of in-home sensor systems could accommodate this desire and maintain safety. Different types of contactless sensors can be integrated into the homes’ infrastructure, for example, motion, temperature, pressure or bed sensors, and infrared imaging. These sensors enable the monitoring of activities of daily living of older adults such as personal hygiene, toileting, cooking, and managing medications. Gradual and acute changes can be detected by these sensors and shared with professional and informal caregivers allowing them the opportunity to provide assistance or early intervention. There has been long-standing interest surrounding in-home sensor systems for older adults as this technology could support their ability to live independently and delay institutionalization [45-48]. However, with the recent global COVID-19 pandemic, in-home sensor systems may have a secondary benefit. Sensor-based health parameter data could provide evidence for developing recommendations around when home confinement is necessary and when the adverse effects of staying home (eg, social isolation, decreased physical exercise, and poor mental health) outweigh the benefits. Such recommendations could provide support for evidence-based clinical decision-making.

This study has important implications for individuals providing care support to older adults aging in place. Study findings suggest an opportunity to use remote sensing technologies in the ongoing monitoring of community-dwelling older adults, particularly in times when quarantine and social distancing measures are in place. Previous research suggests that older adults living with embedded, sensor technologies found a sense of comfort and security in having the technologies installed, and a desire for those providing care support to view the data [49]. Strides have been made over the past 10 years to create a consumer-facing interface for older adults and their caregivers to access the sensor-generated data [50]. Individuals who provide care support to the aging population can review sensor-generated data to observe changes in activity levels and physiological functioning. Trends in the data over time can help inform early, at-home interventions. For instance, in this study the increase in restlessness in bed and time spent in bed could provide an opportunity for a conversation about sleep hygiene and mental health. Interventions to engage the older adult in activities that promote cognitive stimulation could be negotiated between the older adult and their caregiver. Social engagement activities can also be a point of intervention, even during quarantine and social distancing, such as video conferencing with loved ones and attending various group activities internet-based or remotely. Participation in such activities during the COVID-19 pandemic provided older adults with increased entertainment and mental stimulation [51].

### Limitations

While our secondary analysis using remote sensors reveals significant findings about the impact of confinement on older adults, it is important to recognize some inherent limitations in our methodology. First, the dependence on observable metrics might have hindered fully capturing the range of subtleties associated with older adults’ adherence. The nuances of individual and facility staff compliance, the variances in the use of social distancing techniques, or the degrees of adherence within particular demographic groups, for example, may not be fully captured by our assessment. Second, the unprecedented circumstances of the COVID-19 pandemic presented challenges in maintaining sensor networks, particularly for motion activity tracking. The need for manual battery replacements disrupted continuous data collection, potentially affecting the accuracy and reliability of insights derived from motion sensors. Third, while the behavioral markers used in the study are supported by previous research and established methodologies, their validation within the specific context of this cohort remains a limitation. Future studies should prioritize incorporating such validation measures to ensure the reliability of identified markers. Fourth, the sample consisted solely of residents from senior living facilities in mid-Missouri, which may limit the generalizability of the findings. This may limit the generalizability of the findings, as the sample may not adequately represent older adults residing in different geographic regions or those with diverse socioeconomic backgrounds with disparities of technology access in mind. As such, this limitation should be considered when drawing conclusions from the study’s findings. Fifth, the limited exploration of the psychological impacts of isolation, with a focus on basic physical and mental health metrics, highlights the need for future studies to incorporate qualitative data, such as patient interviews or psychological assessments, to gain a more nuanced understanding of participants’ mental health. Finally, while precautions are taken in ensuring accurate sensor measurements, the introduction of noise from pets and visits does occur and analysis was conducted on the daily aggregation of sensor measurements to help account for this noise. These limitations underscore the need for enhanced validation strategies, broader participant diversity, and more comprehensive data integration in future research.

A recent systematic review discerns the bidirectionality between loneliness and the functional trajectory of aging older adults [52]. This observation emphasizes the need for further investigative endeavors aimed at identifying the latent confounders affecting these phenomena. To systematically identify these determinants, the acquisition of a continuous, high-fidelity data stream is imperative. In this regard, the deployment of remote passive sensors emerges as a potent modality, while ensuring minimal disruption to aging in place older adults. In future research endeavors, the implementation of causal inference analyses on the dataset obtained through the aforementioned methods holds promise for uncovering the intricate causal foundations that underlie, the complex framework of aging in place among older adults [53].

### Conclusions

This study presents important implications for the health and well-being of aging in place older adults, particularly during times of home confinement. The results suggest the potential value of increased support and interventions to help maintain physical and cognitive health, while also indicating the importance of tailoring these interventions to the unique needs and circumstances of each individual. By recognizing and addressing the unique challenges faced by older adults, we can design interventions that effectively support physical, cognitive, and emotional health.

## Abbreviations

ACSM: American College of Sports Medicine
AHA: American Heart Association
CMS: Centers for Medicare and Medicaid Services
PIR: passive infrared
WHO: World Health Organization
